# Kinect as a Tool for Gait Analysis: Validation of a Real-Time Joint Extraction Algorithm Working in Side View

**DOI:** 10.3390/s150101417

**Published:** 2015-01-14

**Authors:** Enea Cippitelli, Samuele Gasparrini, Susanna Spinsante, Ennio Gambi

**Affiliations:** Dipartimento di Ingegneria dell'Informazione, Università Politecnica delle Marche, Via Brecce Bianche 12, Ancona 60131, Italy; E-Mails: s.gasparrini@univpm.it (S.G.); s.spinsante@univpm.it (S.S.); e.gambi@univpm.it (E.G.)

**Keywords:** depth sensor, side view, markerless joint estimation, trajectory estimation, Tinetti test

## Abstract

The Microsoft Kinect sensor has gained attention as a tool for gait analysis for several years. Despite the many advantages the sensor provides, however, the lack of a native capability to extract joints from the side view of a human body still limits the adoption of the device to a number of relevant applications. This paper presents an algorithm to locate and estimate the trajectories of up to six joints extracted from the side depth view of a human body captured by the Kinect device. The algorithm is then applied to extract data that can be exploited to provide an objective score for the “Get Up and Go Test”, which is typically adopted for gait analysis in rehabilitation fields. Starting from the depth-data stream provided by the Microsoft Kinect sensor, the proposed algorithm relies on anthropometric models only, to locate and identify the positions of the joints. Differently from machine learning approaches, this solution avoids complex computations, which usually require significant resources. The reliability of the information about the joint position output by the algorithm is evaluated by comparison to a marker-based system. Tests show that the trajectories extracted by the proposed algorithm adhere to the reference curves better than the ones obtained from the skeleton generated by the native applications provided within the Microsoft Kinect (Microsoft Corporation, Redmond, WA, USA, 2013) and OpenNI (OpenNI organization, Tel Aviv, Israel, 2013) Software Development Kits.

## 1. Introduction

The “Get Up and Go Test” (GUGT), also called the “Tinetti test” [1], is used as an assessment tool to evaluate a subject's capabilities in gait and balance. The test is typically performed in hospital environments and consists of standing from an armless chair and starting to walk. Nowadays, the execution of the test is evaluated by qualified medical staff, who fill in a table with scores. Obviously, those scores are partly subjective, as they depend on the different level of expertise of the operator who performs the assessment. Furthermore, in order to get an unbiased outcome at different times, the test should be evaluated always by the same operator. With the aim of providing a more objective assessment procedure, which can be repeated at different times, it might be useful to design an automatic system that outputs an unbiased (*i.e.*, repeatable) score of the test, based on the use of the Microsoft Kinect sensor, which integrates depth and color cameras. At present, Kinect is the cheapest depth sensor available on the market, compared to, for example, a time of flight (TOF) camera; at the same time, it is able to provide comparable performances in short range environments [2,3]. One of the most interesting features of the sensor is the possibility of extracting skeleton joints from the depth data captured. The joint estimation algorithm developed by the Microsoft Research Team [4] is natively provided with the sensor software development toolkit (SDK). In addition to the Microsoft SDK software (Microsoft Corporation), the depth information captured by the Kinect sensor can be processed also by alternative tools, such as the OpenNI (Open Natural Interaction) SDK [5]. This software environment, developed by PrimeSense, uses the NITE middleware to implement the automatic joint estimation and tracking process.

The available algorithms mentioned above perform very well when the subject is in a frontal view with respect to the sensor, *i.e.*, an operational condition that is typical of gaming, which is indeed the target application for which the sensor was designed. Unfortunately, the joint extraction capability of the sensor, enabled by the algorithms currently available, becomes unreliable when the subject is in a side view (left or right) with respect to it. Moving from the assumption that a reliable tool for the automatic assessment of the GUGT should be able to work either in a frontal or in a side configuration [6], in this paper, we propose a new algorithm to automatically estimate the positions of the joints in the skeleton, from the depth information provided by Kinect, when the subject is in a side view with respect to it. Once the joints are located, the algorithm is able to track them in a sequence of depth frames captured during the GUGT execution. The aim of our research is to keep the complexity of the algorithm very low, so that it could be potentially applied in real time. In fact, compared to the typical computational complexity and training phase required by machine learning approaches, *i.e.*, [7–9], usually adopted to solve feature extraction problems, the proposed algorithm only needs to gather some preliminary information about the subject and the environment, before running the test. The solution herein discussed extends the approach preliminarily outlined in [10] and relies on the following main steps:

- construction of a background depth frame (*BF*): the *BF* captures the test environment, without the subject;
- analysis of the front-plane pose: the person is located in the front-plane pose with respect to the sensor. In detail, the subject is oriented towards the sensor standing at a fixed distance from it, with outstretched arms and open hands. This configuration is useful to detect the joints and to compute the distances between adjacent ones, based upon anthropometric models [11]. The information collected at this step is used in the following trajectory estimation phase;
- trajectory estimation phase: the subject performs the GUGT, and the proposed system identifies and tracks the joints at each captured frame. The interpolation of the discrete coordinates of each joint provides the continuous trajectory associated with the movement.

**Figure 1.**
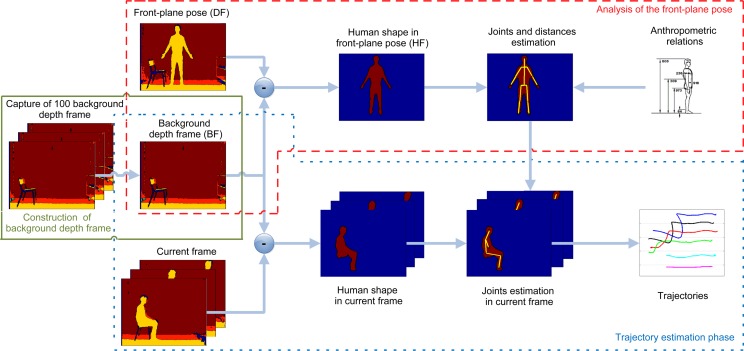
Overview of the proposed joint estimation algorithm's main steps.

**Figure 2.**
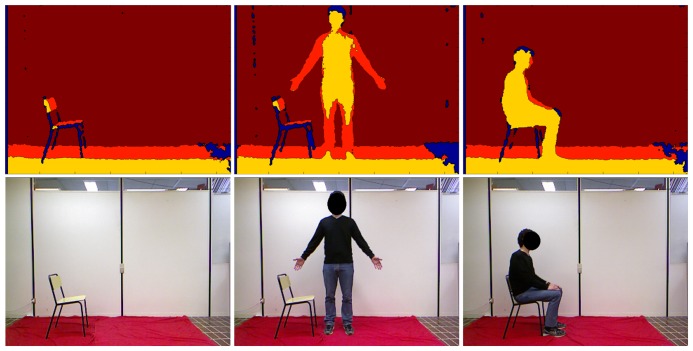
Depth maps and RGB images of the three main steps of the algorithm. (**Left**) Background construction; (**middle**) front-plane analysis; (**right**) “Get Up and Go Test” (GUGT) tracking (start position).

The entire procedure is sketched in Figure 1, in which every step described above is highlighted in boxes. With respect to the preliminary work presented in [10], this paper provides first a detailed description of all of the steps included within the proposed system, followed by a thorough discussion of the results obtained through experimental tests and validation. For better clarity, Figure 2 shows depth maps and corresponding pictures in the RGB domain, related to the three main phases performed by the algorithm. Starting from the left, depth and RGB frames related to the background construction, front-plane analysis and GUGT tracking steps, respectively, are presented.

The system estimates the coordinates in the depth frame domain, of up to six joints captured from a side view: head, shoulder, elbow, hip, knee and ankle. The reliability of the proposed solution is evaluated by comparing the trajectory of each joint within subsequent depth frames, to a ground truth system that provides reference trajectories. The reference trajectories associated with each joint are derived from a marker-based system, working on the Kinect infrared (IR) channel data, that locates and tracks the sticky square markers of 2-cm side, applied on the body of the subject, that are active in the IR domain. A similar comparison is also used to validate the joints estimation algorithm embedded within the Kinect SDK and the one provided by OpenNI.

The rest of the paper is organized as follows: Section 2 reviews related works and approaches for designing automated modalities for the GUGT assessment, whereas the proposed method is detailed in Section 3. Section 4 presents the validation methodology applied to the different algorithms that are evaluated; corresponding results and a related discussion are provided in Section 5. Finally, Section 6 concludes the paper.

## 2. Related Works

From the analysis of the state of the art in the design of automated modalities for the GUGT evaluation, a number of heterogeneous techniques and markerless joint estimation algorithms emerge. In [12], the authors propose the use of accelerometers and gyroscopes to measure the angles between hip, knee and ankle and to derive the corresponding temporal curves. The major drawbacks of this approach are the use of wearable sensors (with related acceptability issues by the subject) and the need for an initial calibration. Further, the proposed approach does not provide any information about the upper limbs. In [13], the location and analysis of the joints are obtained by tracking sticky markers pasted on specific points of the subject's body in a captured video sequence. The advantage of this solution consists of a simpler data analysis than the previous technique and a less invasive shape for the markers, but again, the subject has to wear foreign objects (*i.e.*, the markers) to feed data into the acquisition system. In the last few years, the availability of inexpensive depth cameras has encouraged their use to develop markerless tracking solutions. Many works, such as [14–17], are focused only on upper body joint estimation. Ye *et al.* [18] present an accurate algorithm to estimate the position of 19 joints located on the body, but due to the complexity of the model adopted to represent the person, the system needs to run offline. In [19,20], the authors take advantage of the coordinates automatically computed by the sensor SDK, which, however, dictates specific hardware/software requirements. The solution herein presented, on the contrary, can run on multiple platforms and operating systems, which support different Kinect libraries [5,21]. With respect to the related work discussed in this section, the proposed algorithm uses neither a machine learning approach, nor the native Microsoft SDK skeleton data, nor equivalent graphical engine systems. The proposed solution exploits raw depth data and anthropometric relationships to infer the joint positions on a frame-by-frame temporal basis.

## 3. The Proposed Method

This section describes the whole architecture of the algorithm proposed for the estimation of the joint position from the side depth view of the subject.

### 3.1. Construction of Background Depth Frame

The system setup for the joint extraction in side-view is composed by a Microsoft Kinect sensor elevated at a 92-cm height from the ground and at a distance of 330 cm from a wall. These values represent a tradeoff between pixel density and sensor coverage area according to the recommendations for use provided by the sensor manufacturer. Locating the sensor closer to the wall may improve the performance of the joint estimation phase, because the pixel density increases. The drawback of this option is the reduction of the sensor coverage area, so the height of people admitted to the test must be limited to a given value. Increasing the distance among the sensor and the wall could enlarge the coverage area, but this leads to a reduction of pixel density and, consequently, an increasing error in joint estimation. The configuration chosen in this work allows the complete inclusion of the subject inside the scene, also when he/she stands, near to the sensor. A carpet is used to cover the floor and to reduce the disturbances in depth estimation, due to light reflections from the floor. With the goal of mitigating the variability of depth data, 100 consecutive depth frames are captured at a rate of 30 fps (frame resolution: 320 × 240 pixels), to create a single reference background frame (*BF*), by averaging each pixel value in time. The armless chair needed to perform the GUGT, located at a distance of 30 cm from the wall, is the only object present in the scene during the background frame acquisition. The chair should preferably remain fixed in the same position during the test execution. If the chair is slightly moved, for example during the get up phase, some noise can remain after the background subtraction step performed by the algorithm, due to frame mismatches.

### 3.2. Analysis of the Front-Plane Pose

For the acquisition of the front-plane pose, the subject is placed approximately at a distance of 3 m from the sensor, as shown in the “front-plane pose” depth frame in Figure 1. Only a single depth frame (*DF*) is captured in this condition, with the aim of estimating the anthropometric relationships among the most important parts of the subject's body (upper/lower arm, femur, tibia, head, *etc.*). Let *DF*(*x*, *y*) be the depth information at column *x* and row *y* in the *DF* (*i.e.*, the “pixel” in the *DF* located at position (*x*,*y*), where 0 ≤ *x* ≤ 320 and 0 ≤ y ≤ 240) and *BF*(*x, y*) the equivalent in the *BF*. Equation (1) describes the mathematical relation used to generate the foreground frame (*FF*) value in (*x, y*):

(1) $$FF\left(x,y\right)=\{\begin{array}{ll} 0, & \text{if}\;|DF\left(x,y\right)-BF\left(x,y\right)|<Th\\ 1, & \text{otherwise} \end{array}$$

The quantity *Th* represents a depth threshold (set at a value of 150 mm) to distinguish foreground pixels from background ones. This value is independent of the wall-sensor distance and is derived experimentally, assuming a 30-cm distance of the subject from the wall. Such a threshold has been chosen to correctly recover the lower part of the human subject in the depth map. In fact, the pixels corresponding to the subject's feet have a depth value near to the depth value of the floor pixels, so a quite low threshold must be set to distinguish these pixels from the background.

The “human shape in front-plane pose” (*HF*) frame detailed in Figure 1 shows the pixels located along the human silhouette, which is the biggest object revealed by the algorithm that finds connected components in the frame.

**Figure 3.**
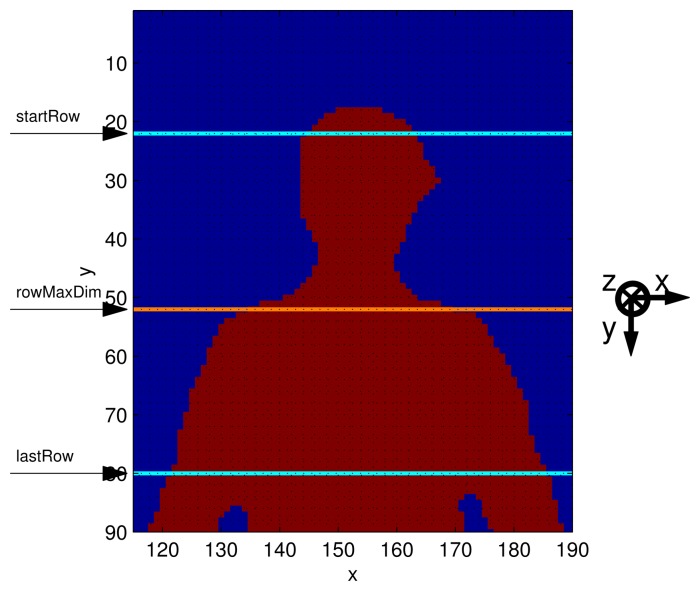
Zoom of the upper body section of human shape. *startRow* and *lastRow* identify the frame area where the algorithm tries to locate the *rowMaxDim*, which represents the top of the shoulder.

Once the human silhouette has been identified, the algorithm locates the joints in the side-view by using anthropometric models, then it computes the distances between some of them. Using heuristic numerical coefficients, the models describe the geometrical relationships between different parts of the body. Thanks to the rigidity of the skeleton structure, it is reasonable to assume that the same relations are maintained, even during the execution of movements. At first, the software analyzes the *HF* frame to identify the head joint, through the following steps:

1. the parameter *human Height* is evaluated, by subtracting the indices of the bottom and top rows of pixels belonging to the human silhouette;
2. the first position from which the head joint search has to start (named *start Row*) is located by subtracting 100 mm from the region corresponding to the first row of the silhouette in *HF*. The relation between the real-world coordinates system [*X_d_ Y_d_ Z_d_*]*^T^* (given as mm) and the depth frame coordinates system [*x_d_ y_d_* 1]*^T^* (given as pixels) is computed using the calibration parameters of the Kinect depth camera through Equation (2) [22]:
  (2) $$\left[\begin{array}{c} X_{d}\\ Y_{d}\\ Z_{d} \end{array}\right]=K_{d}^{-1}\left[\begin{array}{c} x_{d}\\ y_{d}\\ 1 \end{array}\right]$$
  where *K_d_* is the matrix that contains the intrinsic parameters of the depth camera. Equation (2) allows the translation of anthropometric relations, given in the 3D domain, into rules valid in the depth frame domain, where quantities are given in pixels;
3. the number of columns belonging to the human shape, on the analyzed row, is computed. The process goes on, increasing the row index by steps of 40 mm; the magnitude of the steps has been optimized to be able to detect the different width of the neck and shoulders. The process stops when the index difference between *startRow* and the last row analyzed equals one third of *humanHeight* (defined as *lastRow*). Such a lower bound (*lastRow*) has been chosen to limit the computation necessary to identify the shoulders, based on the assumption that they are necessarily located in the first (upper) third of the human silhouette. This step is shown in Figure 3;
4. the difference between the amounts of columns belonging to the nearest rows processed is computed;
5. the row featuring the maximum difference is called *rowMaxDim*, and it identifies the top of the shoulders (Figure 3);
6. the quantity *shiftRow* is defined as the difference between *rowMaxDim* and the first row that belongs to the human shape, divided by three;
7. *shiftRow* must be added to the first row of the human shape, to find the *headRow*, which represents the *y*-coordinate of the head joint;
8. the corresponding *x*-coordinate is obtained by averaging the first and the last indices of the columns that belong to the human shape, in the *headRow*.

A shift of 40 mm below *rowMaxDim* identifies the row at which the shoulders are located. Once the first column occupied by the human silhouette is identified, another horizontal shift of 40 mm is applied to the row corresponding to the shoulders, to locate the *x*-coordinate of the left shoulder inside the human shape. Finding the hip joint *y*-coordinate requires evaluating the head height. This quantity is obtained as the difference between the first row containing the human shape and *rowMaxDim*, reduced by 100 mm, which models the neck height. The head height must be multiplied by a coefficient *c* to find a row shift that identifies the *y*-coordinate of the hip, from the first row containing the human shape. This coefficient depends on the ratio between the head height and the total height of the human subject (*humanHeight*). Let *R* be the ratio between *humanHeight* and the height of the human head; the coefficient *c* is estimated using data in [11]. We extracted some datasets from [11], whereby all of the necessary measurements are available.

The datasets are identified by a number, *i.e.*, 7 for the “AIR FORCE WOMEN- 1968” set, 51 for the “ITALIAN MILITARY-1961” set, and so on. The first column in Table 1 lists the datasets considered. For each set, the ratio *R* is evaluated considering the Stature (Number 805 in [11]) and the height of the head (Number 595); results are in the fourth column of Table 1. The quantity Buttock Height (Number 188) represents the distance of the hip joint from the floor; therefore, the sixth column, obtained by subtracting the Buttock Height to the Stature value, collects the values of the distance between the top of the head and the hip joint. The coefficient *c* is given by the ratio between the quantity in the sixth column and the height of the head. By relating the quantity *R* and the value of *c* in Table 1, the following conditions are obtained:

(3) $$c=\{\begin{array}{ll} 3.2 & R<6.4\\ 3.4 & 6.4\leq R<7.2\\ 3.6 & 7.2\leq R<7.5\\ 3.8 & 7.5\leq R<8\\ 4 & 8\leq R<8.8\\ 4.2 & R\geq 8.8 \end{array}$$

The estimation of the lower part of the body starts from the ankle joint row. Such a row is found by decreasing the index of the last row of the human shape by 40 mm, in order to locate the ankle inside the body.

The evaluation of the distance between ankle and knee is based on an anthropometric ratio, *i.e.*, the height of the human is multiplied by a coefficient of 0.2. This coefficient is also obtained from [11] using the following measurements: Knee Height Sitting (529), Medial Malleolus Height (579) and Popliteal Height (678).

The remaining joints are located by searching the left arm in the frame, and the process goes on as described below:

1. the algorithm extracts the left part of the *HF* frame, looking for a gap in the column indices between the left arm and the body, along the row where the hip is located;
2. in this sub-frame, the last row index that identifies the end of the arm can be found;
3. an upward row shift by half the head height identifies the row at which the left wrist is located;
4. averaging the column indices of the sub-frame belonging to the row where the wrist joint is located gives the *x*-coordinate of the wrist joint itself;
5. once the distance between the wrist and the shoulder is computed, the elbow can be located in the middle of this range.

The following distance information, head-shoulder (*HS*_*dist*), shoulder-elbow (*SE*_*dist*), elbow-wrist (*EW*_*dist*), ankle-knee (*AK*_*dist*) and knee-hip (*KH*_*dist*), is stored and used in the next steps of the algorithm.

### 3.3. Trajectory Estimation Phase

For each input depth frame, the algorithm finds the human shape through the technique described in the previous section.

The head joint is located by using the shift information derived from the frame showing the front-plane pose, starting from the first non-null row. The corresponding column is the average of the pixel column indices that belong to the human silhouette, in the same row.

The remaining joints are located by analyzing only the side of the human silhouette in the depth frame closest to the sensor. The side selection can be obtained through the following steps. Starting from the lowest row of pixels associated with the subject's shape, the algorithm applies an up-shift of a number of rows equal to half the value of *AK*_*dist*, and catches the index of the column featuring the lowest depth value. Around this pixel, a square sub-matrix of the *AK*_*dist* side is selected, with the aim of mitigating fluctuations due to noise. In this region, all of the pixels, for which the difference of their depth value from the depth value of the central pixel overcomes the *sideManThreshold* (80 mm), are eliminated. By taking into account the remaining pixels, the average depth value is calculated. Based on this distance, the side part of the human shape is estimated, as the set of all pixels located around an 80-mm gap region.

The shoulder joint can be found by initially assuming a vertical alignment with the head and a row-gap of *HS*_*dist*. Once the row is detected, the corresponding column is computed, as the average of the first and the last indices of the columns that belong to the human shape, in the *shoulderRow*. The estimation is then improved: the head-shoulder angle is derived, and the segment connecting the joints needs to be equal to *HS*_*dist*. This expedient is useful to maintain the shoulder joint always near its “real” position, especially when the subject stands up during the GUGT execution.

**Figure 4.**
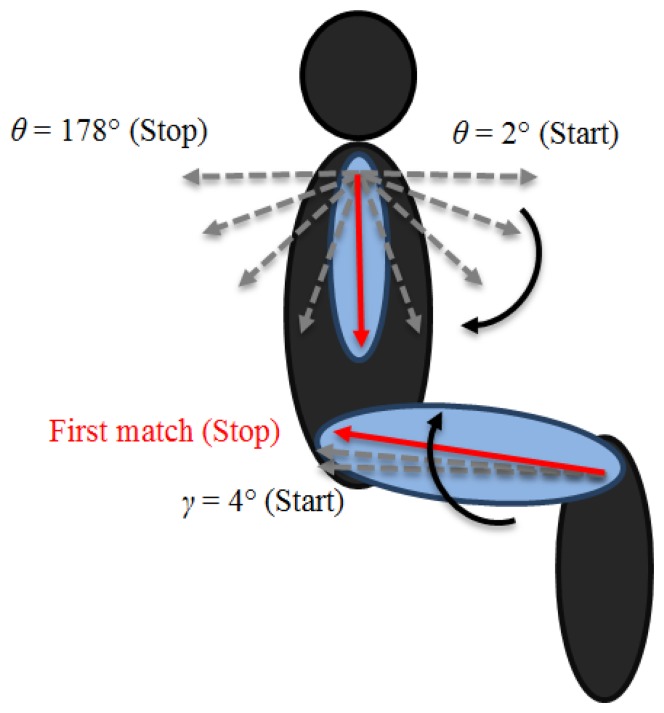
Elbow and hip identification procedure. (**Top**) Rotating vector used to estimate the elbow position; (**bottom**) the vector that identifies the hip coordinates.

To locate the elbow joint, it is necessary to select only the pixels around the arm. We follow the same approach used to extract the side of the human shape, with the sub-matrix centered *SE*_*dist*/2 rows below the *shoulderRow*, the same column index of the shoulder. Starting from the shoulder joint, a vector of length *SE*_*dist* is rotated from *θ* = 2° to *θ* = 178°, as shown in Figure 4. At each step, the algorithm calculates the number of overlapping pixels between the vector and the arm region extracted. The correct position of the elbow corresponds to the maximum number of matching pixels.

The estimation of the lower part of the body is independent from the upper one and can be calculated separately. The process adopted to locate the ankle joint follows the same reasoning used for the head joint, but in this case, the algorithm starts counting from the last row index of interest. The knee joint positioning uses the same idea applied to the shoulder joint, which consists of a first vertical identification of the *kneeRow* by *AK_dist* and a subsequent adjustment, by considering the angle between knee and ankle. The estimation of the hip joint consists of a vector, anchored in the knee joint. The vector of length *KH_dist* starts rotating from *γ* = 4° to the best matching angle by 2° steps, as shown in Figure 4.

**Figure 5.**
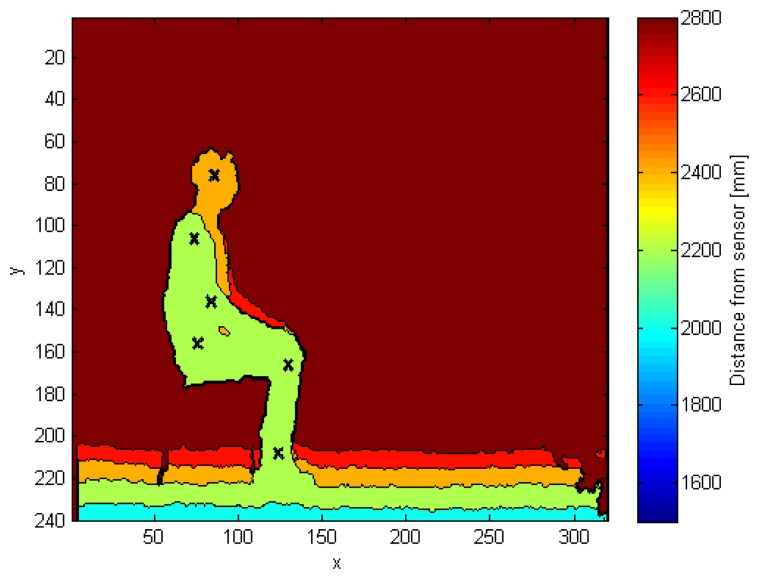
Side-view depth frame, where the six estimated joints are positioned.

Figure 5 shows the side view of the human shape in the depth map, in which every cross represents a different joint. Starting from the top, we can identify the head, shoulder, elbow, hip, knee and ankle joints.

## 4. Algorithm Validation

**Figure 6.**
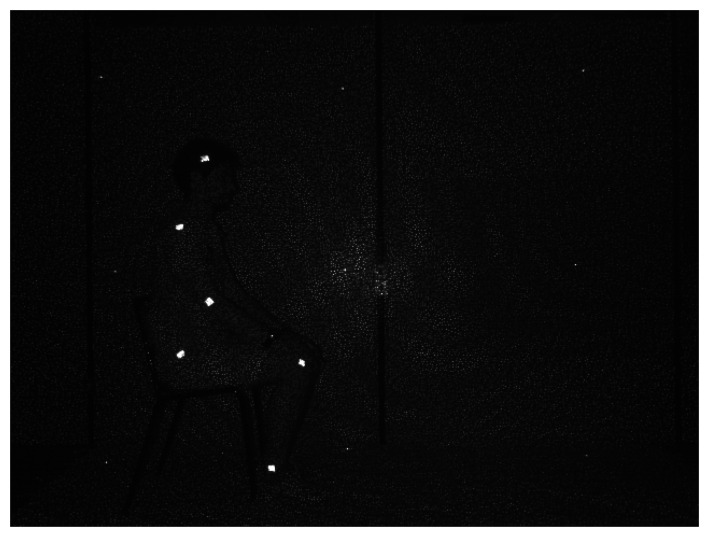
Infrared frame captured by the Kinect sensor.

The validation of the proposed algorithm for joint location and tracking during the GUGT has been performed by evaluating the adherence of the estimated joint trajectories to the real trajectories, obtained through a marker-based system in the domain of pixel coordinates in a frame. More in detail, since the Microsoft Kinect sensor allows capturing IR data, IR active sticky markers have been used to identify the real joints in the human body. Figure 6 shows an IR frame, captured when the subject is seated on the chair and ready to perform the test. It is easy to identify the positions of the six considered joints: head, shoulder, elbow, hip, knee and ankle. The performances of the proposed algorithm are evaluated in the frame domain, through the pixel coordinate mismatch between the position of each joint obtained by the marker-based algorithm and the position of the corresponding joint, evaluated by the proposed solution. The trajectories of the same joints acquired by tracking a set of markers in a video-based system are considered as a reference; the algorithms provided by Microsoft Kinect for Windows (Algorithm 1) and OpenNI SDK (Algorithm 2) represent, respectively, a second and a third system, against which the algorithm we developed (proposed) can be compared.

Tests have been performed in a lab premise, but the same setup may be reproduced in any other indoor environment, assuming the aforementioned distance conditions are respected. The validation experiment starts with the creation of a background depth frame (*BF*), as described in the Sub-Section 3.1, which means that the chair used for the test is the only object in the scene. The second phase concerns the analysis of the front plane pose, which consists of the capture of a depth frame where the subject is standing in front of the sensor, at an approximate distance of 3 m. At this step, the algorithm subtracts the background and gets the human shape, which is processed to compute the distances among the parts of the subject's body. The last step consists of the real GUGT execution, and to validate the algorithm, the IR active sticky markers that identify the joints are applied on the subject. When the subject is seated on the chair and ready to perform the test, the operator starts the data acquisition. During the GUGT execution, the subject stands up and steps forward along a distance of a few meters. The operator stops the process of data acquisition when the subject is standing on his sagittal plane with respect to the sensor. An *ad hoc* acquisition tool has been developed to capture multiple data from Kinect simultaneously. This software exploits Kinect for Windows SDK 1.7 to capture and store the following data streams:

- depth frames, used as inputs to the proposed algorithm and to Algorithm 2;
- IR frames, used to recover the real joint positions identified by markers;
- skeleton frames, computed by Algorithm 1.

**Figure 7.**
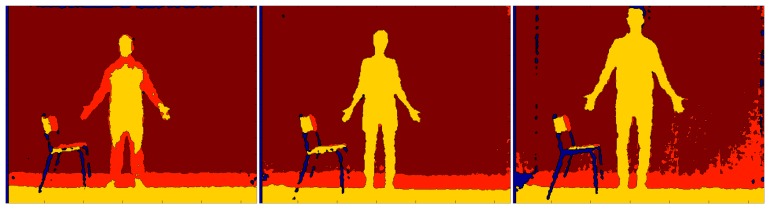
Depth maps of three subjects, featuring the different body sizes involved in the test.

The proposed algorithm has been evaluated on 18 GUGT executions by different subjects of heights ranging from 1.6 to 1.85 m and with different body sizes. A sample set is provided in Figure 7.

## 5. Results and Discussion

An objective method to compare the trajectories of the four tracking systems relies on evaluating the Euclidean distance among the joint coordinates in the frame domain of dimensions 320 × 240 pixels. Defining by (*x_i_*,*_k_*,*_m_*,*y_i_*,*_k_*_,_*_m_*) the pixel coordinates of the *i*-th joint in the *k*-th frame identified by the marker-based method (*m*) and by (*x_i,k_*_,_*_p_, y_i,k,p_*) the same coordinates obtained by the proposed algorithm (*p*), it is possible to compute the magnitude of the difference vector, *D_i_*_,_*_k_*:

(4) $$D_{i,k}=\sqrt{{\left(x_{i,k,m}-x_{i,k,p}\right)}^{2}+{\left(y_{i,k,m}-y_{i,k,p}\right)}^{2}}$$

By repeating the computation for the entire set of depth frames (*K*), we define a vector *D_i_* referring to each *i*-th joint:

(5) $$D_{i}=\left[D_{i,1,}\;D_{i,2,}\ldots \;D_{i,k}\right]\;,\;i=1,2,\ldots 6$$

The same sets of difference values are computed between the pixel coordinates provided by the marker-based method (marker), Algorithm 1 and Algorithm 2, respectively. Each Di gives the values of the pixel coordinate differences of the *i*-th joint location, at each frame, provided by the different algorithms; its average (*μ*) and standard deviation (*σ*) may be assumed as global performance parameters of the algorithm. In order to increase the relevance of the statistical indices, the offset between each markerless trajectory and the marker-based coordinates has been compensated before computing the *D_i_* vectors.

**Figure 8.**
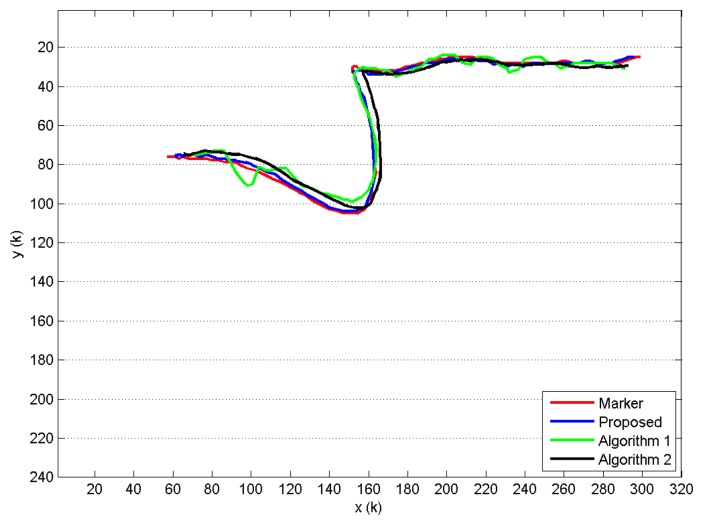
Head joint trajectories provided by the four compared algorithms. The trajectories are provided by tracking the joint (*x, y*) pixel coordinates in each *k*-th frame of dimensions 320 × 240. The amount of frames considered is *K* = 120.

**Figure 9.**
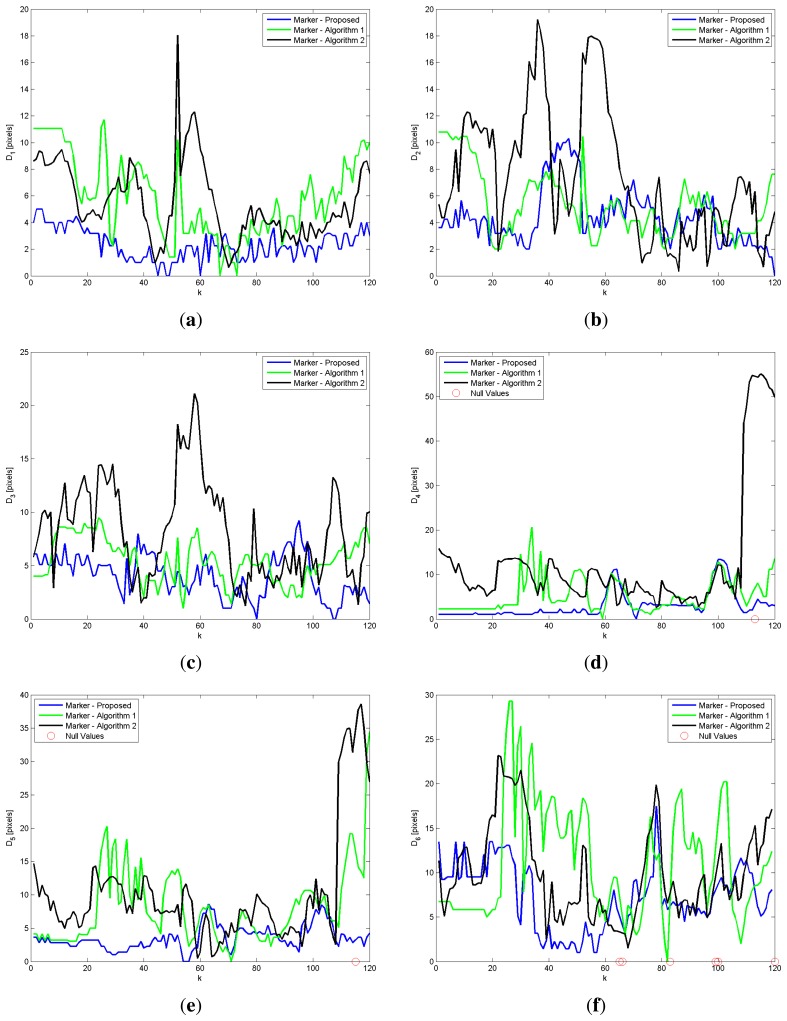
Amplitude values of the difference vectors *D_i_* computed between the trajectories provided by the marker-based and the markerless systems, for: (**a**) head joint; (**b**) shoulder joint; (**c**) elbow joint; (**d**) ankle joint; (**e**) knee joint and (**f**) hip joint evaluated over 120 depth frames.

When compared to Algorithm 1 and Algorithm 2, the proposed approach shows a better trajectory estimation. This result is visible in Figure 8, where the trajectories revealed for the head joint of a single GUGT execution and *K* = 120 depth frames captured are shown. The curve named proposed, provided by the proposed solution, compared to the Algorithm 1 and Algorithm 2 ones, better approximates the movement captured by the marker-based system (see Figure 6), named marker. To improve the visual understanding, all of the trajectories in Figure 8 have been unbiased by removing the fixed offset derived from the least squares computation. Figure 9 shows the differences *D_i_* provided by the markerless systems, with respect to the reference one (marker-based), for all of the joints estimated during the same GUGT execution over *K* = 120 frames. The better head joint estimation given by the proposed solution is confirmed in Figure 9a, denoted by smaller values and reduced variability of the vector *D*_1_ (marker-proposed curve).

The algorithm proposed is closer to the ground-truth one, because it does not suffer from the fluctuations that affect the other two joints estimation systems. In particular, Figure 9b shows some peaks around Frame 35 of the Marker-Algorithm 2 curves. Around this frame, the subject starts to get up from the chair, and he is tilted forward. Figure 9d–f shows the same variability problem in the Marker-Algorithm 1 curve. A similar behavior happens for ankle and knee joints (Figure 9d,e) in the last frames of the Marker-Algorithm 2 curve.

It may happen that an IR marker used for validation gets hidden from the sensor at a given *k*-th frame, due to partial occlusion caused by the movement of the subject. If this condition happens, the vector *D_i,k_* cannot be evaluated, and it takes a null value. This phenomenon can be seen in Figure 9d–f. Obviously, the frames that show a null difference vector are excluded from the computation of the statistical values *μ* and *σ*.

Table 2 shows the statistics of the different algorithms evaluated against the marker-based one, for the same GUGT realization. The proposed system outperforms Algorithm 1 and Algorithm 2 in the average and standard deviation, for all of the joints captured in the side-view. The proposed approach shows the best performance for the standard deviation of the head joint (1.1426), while the hip joint has the highest average value and variability in the set of joints analyzed.

**Figure 10.**
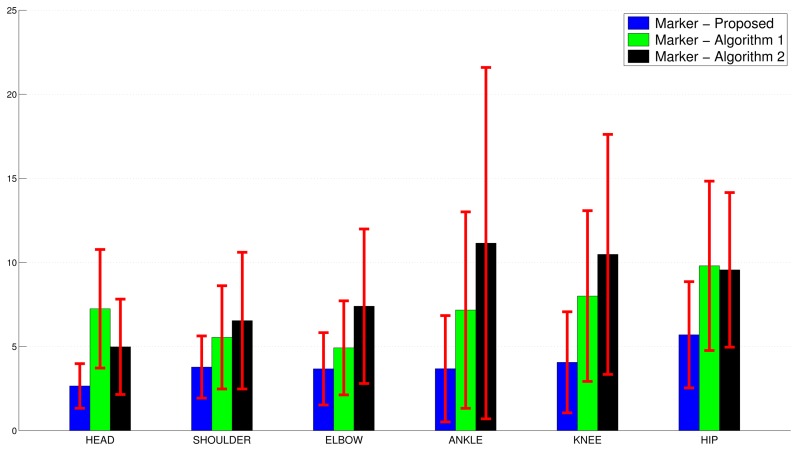
Performance comparison of the proposed algorithm, Algorithm 1 and Algorithm 2 *vs.* the marker-based solution. The average mean and the average standard deviation values of the Euclidean distances among trajectories are shown.

Figure 10 shows the global performance parameters *μ* and *σ* for each joint, averaged over 18 GUGT executions.

As a final remark, the computational requirements of the algorithm working at full depth frame resolution have been estimated, by evaluating the execution time of a MATLAB implementation running over 2800 depth frames. On an Intel i7 Windows 8.1 and 8GB RAM desktop PC, the average processing time requested for a single depth frame is 0.051 s. The low computational time requirement exhibited by the non-optimized MATLAB implementation, in conjunction with the fact that a preliminary version of the same algorithm, though working on subsampled depth frames (160 × 120 pixels), has been already effectively implemented in real-time as a C++ code, makes it possible to reasonably assume that a real-time implementation of the full resolution algorithm will be available soon, as well.

## 6. Conclusions and Future Works

An innovative approach to extract the most important skeleton joints from the depth information captured by the Kinect sensor focused on a human subject in side-view has been presented, which can be effectively exploited in a tool for the automated evaluation of Get Up and Go Test executions. The algorithm works on a single depth map and does not apply any machine learning technique. A preliminary depth acquisition of the test environment is needed to perform background subtraction. By comparing the obtained results with the results in [10], a better accuracy has been obtained by processing the frame at 320 × 240 pixels and by aligning the coordinate trajectories before the error computation. Future works will concern an improvement of the estimation strategy to optimize the detection of the hip and elbow trajectories, by taking into account the temporal correlation among the depth frames, and by the execution of a greater number of validation tests. In the future, we will also investigate the suitability of the second generation Kinect sensor, which exploits time of flight technology to provide depth information. Such a technique allows better performances in terms of stability and resolution of the depth frame. A depth frame with a reduced level of noise could improve the performances of the proposed algorithm.

The final goal is to use all of these data of joint positions to derive speed and acceleration information and to provide objective and repeatable evaluations of the Get Up and Go Test. This phase must necessarily be supported by healthcare personnel, able to suggest what the significant parameters that we have to extract from the curves, to obtain relevant posture estimation.

## Figures and Tables

**Table 1.** Estimation values for the *c* coefficient.

**Dataset**	**Stature (*S*) (cm)**	**Head Height (cm)**	***R***	**Buttock Height (*BH*) (cm)**	***S*** − ***BH* (cm)**	***c***
10	161.3	22.56	**7.15**	84.14	77.16	**3.42**
8	162.77	22.08	**7.37**	82.08	80.61	**3.65**
7	162.1	21.91	**7.4**	82.21	79.89	**3.65**
9	161.92	21.76	**7.44**	82.09	79.83	**3.67**
51	170.62	22.31	**7.64**	86.56	84.06	**3.77**
21	174.72	22.45	**7.78**	89.95	84.77	**3.78**
20	177.1	22.4	**7.9**	91.16	85.94	**3.84**

**Table 2.** Statistical parameters associated with differences *D_i_*.

**Joints**	**Proposed *vs.* Marker**		**Algorithm 1 *vs.* Marker**		**Algorithm 2 *vs.* Marker**	
	*μ*	*σ*	*μ*	*σ*	*μ*	*σ*
head	**2.3339**	**1.1426**	5.8878	3.0238	5.5202	2.8346
shoulder	**4.4154**	**2.1392**	5.3514	2.4382	7.4386	4.8854
elbow	**4.0166**	**1.9917**	5.4609	2.0548	8.1691	4.4949
ankle	**3.0386**	**2.9961**	5.2701	3.8576	12.2962	13.1106
knee	**3.5132**	**1.7997**	7.9368	5.706	9.9547	8.1032
hip	**7.2508**	**3.7007**	11.0742	6.3248	10.0549	5.2083
